# Depression as a predictor of postoperative functional performance status (PFPS) and treatment adherence in head and neck cancer patients: a prospective study

**DOI:** 10.1186/s40463-015-0092-4

**Published:** 2015-09-18

**Authors:** Brittany Barber, Jace Dergousoff, Margaret Nesbitt, Nicholas Mitchell, Jeffrey Harris, Daniel O’Connell, David Côté, Vincent Biron, Hadi Seikaly

**Affiliations:** Division of Otolaryngology-Head & Neck Surgery, University of Alberta Hospital, 1E4, Walter Mackenzie Centre, 8440-112 St, Edmonton, AB T6G 2B7 Canada; Department of Psychiatry, University of Alberta Hospital, 1E1, Walter Mackenzie Centre, 8440-112 St, Edmonton, AB T6G 2B7 Canada

**Keywords:** Depression, Head neck cancer, Postoperative functional performance

## Abstract

**Background:**

Head and neck cancer (HNC) is a debilitating disease due in part to its effects on function, including speech, swallowing, and cosmesis. Previous studies regarding depression in HNC have focused on demographic predictors, incidence, and quality of life studies. There is, however, a paucity of studies that objectively address depressive symptoms in HNC patients and the resultant effects on post-treatment functional performance status. The aim of this study was to assess the relationship between preoperative depressive symptoms (PDS) and postoperative functional performance status (PFPS), in addition to other predictors of rehabilitation and survival.

**Methods:**

A prospective cohort study was undertaken at the University of Alberta, including all new adult HNC patients undergoing surgery as primary therapy for HNC from May 2013 to January 2014. Baseline depressive symptoms were measured on the Quick Inventory of Depressive Symptoms (QIDS) questionnaire 2 weeks preoperatively and PFPS was assessed 12 months postoperatively on the Functional Assessment of Cancer Therapy-Head & Neck (FACT-HN) scale. Secondary outcomes included completion of adjuvant therapy, narcotic dependence, return to detrimental habits, loss of follow-up, and length of hospital stay (LOHS). Differences between the Normal-Mild and Moderate-Severe QIDS groups were assessed using Mann–Whitney and Fischer Exact statistical analyses.

**Results:**

Seventy-one patients were included in the study. Mild and Moderate-Severe PDS were 35.2 % and 18.3 %, respectively. Significantly lower FACT-HN scores were noted in the Moderate-Severe group at 12 months (p = 0.03). The risk ratio (RR) for FACT-HN score < 50 % at 12 months in the Moderate-Severe group was 5.66. In addition, significantly lower completion of adjuvant treatment (p = 0.03), significantly higher incidence of narcotic dependence (p = 0.004), and significantly higher LOHS (24 days vs. 18 days; p = 0.02) was observed in the Moderate-Severe group. There was no significant difference in loss of follow-up between the 2 groups (p = 0.64).

**Conclusions:**

The incidence and severity of PDS in HNC patients treated with surgery is high (53.5 %). Patients with Moderate-Severe PDS have significantly decreased PFPS, increased narcotic use, decreased completion of adjuvant therapy, and a longer LOHS. HNC patients should be monitored closely for depressive symptoms.

## Introduction

Head and neck cancer (HNC) is a debilitating disease due in part to its effects on daily patient function including speech, swallowing, and cosmesis. Previous studies have demonstrated that approximately 40 % of patients become depressed in the first year after their diagnosis and treatment for head and neck cancer, and more importantly, this goes unrecognized and untreated [1]. Misono et al. [2] revealed that oral cavity and laryngeal cancer make up 2 of the 4 highest suicide populations amongst cancer patients. These finding may be attributed to the devastating combination of predisposing factors for HNC, incapacitating symptoms, and the sequelae of treatment.

Depression is also a devastating disease robbing patients of function and quality of life. Previous studies regarding depression in HNC have focused on demographic predictors, incidence, and quality of life studies. There is, however a paucity of studies that objectively address depressive symptoms in the HNC patients and their effect on post-treatment functional performance status. Limited literature exists regarding the effect of depression on other factors that may affect rehabilitation and survival, such as completion of adjuvant treatment and return to detrimental habits.

The aim of this study was to assess the relationship between preoperative depressive symptoms (PDS) and postoperative functional performance status (PFPS), in addition to other predictors of rehabilitation and survival.

## Methods

Institutional ethical approval was obtained from the Human Research Ethics Board (HREB) at the University of Alberta. Informed consent was obtained from all participating subjects.

A prospective cohort study of HNC patients presenting to a tertiary cancer care practice at the University of Alberta Hospital was undertaken. The study population consisted of adult patients undergoing major head and neck ablative and reconstructive surgery and adjuvant therapy for a new HNC. HNCs considered eligible for inclusion were mucosal squamous cell carcinoma (SCC), salivary gland tumors, and skin cancers. Thyroid and ocular cancers were excluded from the study given differences in the extent of surgical management for each group. Patients were recruited at the time of preoperative surgical education sessions approximately 2 weeks prior to surgery from September 1, 2013 to March 1, 2014.

### Demographic assessment

Adult patients undergoing surgery and adjuvant radiotherapy for a new HNC were included. Patients with a pre-existing psychiatric history, those who were unable to read or comprehend the questionnaires or lacked capacity to consent, and those unwilling to present for follow-up questionnaires or assessment were excluded. Demographic data regarding age, gender, comorbidities, primary tumor site and stage, pre-treatment substance abuse, and presence of supportive caregivers was collected. Advanced-stage cancers were defined as those tumors with a clinical T-stage greater than 2 or N-stage greater than 0. Patient comorbidities were identified and Charlson Comorbidity Index was calculated for each patient.

### Preoperative depressive symptom (PDS) assessment

Once determined eligible, patients underwent baseline evaluation with the Quick Inventory of Depressive Symptomatology Self-Report (QIDS-SR) questionnaire [3]. This is a self-report, validated questionnaire involving 16 items under the typical 9 domains assessed with regard to depressed mood. This was not utilized as a diagnostic test for depression, but as a screening tool for examining the severity of depressive symptoms. The QIDS-SR is scored from 0 to 27, and patients were classified as normal, mild, moderate, severe (Table 1), with higher scores indicative of more severe depressive symptoms. Previous studies have demonstrated an internal consistency between physician-rated QIDS-SR score and self-report scores as high as 0.94 [4].

**Table 1:** Scoring rubric for QIDS-SR questionnaire as previously published by Rush et al. [3]

QIDS-SR	
Normal	0–5
Mild	6–10
Moderate	11–15
Severe	16–20
Very Severe	>20

*QIDS-SR* Quick Inventory of Depressive Symptomatology-Self Report

The Functional Assessment of Cancer Therapy for Head and Neck patients (FACT-HN) is a multi-dimensional, self-reported assessment of post-treatment functional performance status that was specifically designed for head and neck cancer patients, and has been used extensively in Radiation Therapy Oncology Group (RTOG) trials [5–7]. Social, emotional, physical, family, and well-being domains are addressed, and questions related to these domains are answered on a 5-point Likert scale by the patients. Questionnaires are scored from 0 to 144 with higher scores representative of better functioning. Scores of less than 50 % on the FACT-HN correlate with clinical functional decline [5]. A previous analysis of test-retest reliability regarding the stability of the stand-alone FACT-HN questionnaire has demonstrated an intraclass correlation of 0.89 [8].

### Outcome measures

The primary outcome assessed was the FACT-HN score 12 months postoperatively. Secondary outcomes were those not evaluated in previous literature, including completion of adjuvant therapy (defined as completion of all adjuvant treatments including chemotherapy and radiotherapy), narcotic dependence (defined as persistent use greater than 3 months postoperatively), and loss of follow-up (defined as patients not presenting for 2 consecutive scheduled follow-up appointments with no attempts to reschedule). Return-to-habit status was defined as a postoperative return to detrimental habits, such as tobacco, alcohol, or illicit drug abuse. Length of hospital stay (LOHS) was also calculated for each patient and compared between groups.

After patients were determined eligible for the study, they underwent a baseline QIDS-SR assessment approximately 2–3 weeks prior to surgery during routine preoperative surgical education sessions. Patients were then classified into the Normal-Mild group (0–10) or the Moderate-Severe group (11–27) based on the scores demonstrated in Table 1. Subsequently, the patients underwent resection and reconstructive surgery with routine postoperative care including adjuvant therapy. Follow-up appointments were arranged for 2 weeks, 3, 6, and 12 months postoperatively, as is standard protocol. Patients were reassessed at 12 months postoperatively with both a QIDS and FACT-HN assessment. Secondary outcomes were also assessed at 12 months.

Treatment for depressive symptoms was initiated in the standard clinical fashion, but was not considered part of this observational study. If patients scored in the Mild category, a discussion regarding depressive symptoms was initiated and patients were referred to Psychiatry for assessment upon request. If patients scored in the Moderate or Severe groups, they were referred to Psychiatry for assessment and options for treatment were discussed with the patient. Outcomes of treatment discussion with Psychiatry were recorded and followed for the Moderate-Severe group.

### Statistical analysis

The Normal-Mild group was compared to the Moderate-Severe group regarding FACT-HN scores using a Mann–Whitney analysis. A multiple regression analysis was additionally performed using other known predictors of postoperative functional performance status to assess the predictive value of PDS on PFPS. A risk ratio (RR) was also calculated for a score less than 50 % on the FACT-HN for those patients with Moderate-Severe PDS [5]. Secondary outcomes were assessed using a Fischer Exact analysis as well as a Spearman Correlation analysis to confirm statistically significant findings. LOHS was compared between groups using a Mann–Whitney analysis. Normal-Mild and Moderate-Severe groups were also compared regarding demographic variables, comorbidities, pre-treatment ETOH or illicit drug use, supportive care-givers, and tumor sites, and TNM-staging using Mann–Whitney and Fisher exact analyses. Statistical analysis was performed using SPSS software (SPSS, Version 21.0, Chicago, IL).

## Results

Seventy-five patients were approached for recruitment to the study; 1 refused participation, 2 did not fully complete the baseline QIDS questionnaire, and 1 did not undergo adjuvant therapy. The compliance rate for baseline PDS assessment was thus 96 % (72 of 75 patients). Of the 71 patients eligible for participation, 58 patients (81.7 %) scored in the Normal-Mild range upon baseline assessment, with the remaining 13 (18.3 %) scoring in the Moderate-Severe group.

Initial examination of demographic data for the entire cohort revealed findings typical of the HNC population, with a mean age of 59.7, a male gender predominance (70.4 %), and primarily advanced-stage disease (90.1 %). The Charlson Comorbidity Index (CCI) for the entire cohort was 70.2 %. Further examination of each study group revealed no differences in mean age, gender, TNM staging, cancer site, reconstruction, adjuvant therapy, pre-treatment substance abuse, presence of supportive care-givers, or Charlson Comorbidity Index between the Normal-Mild and Moderate-Severe groups (Table 2).

**Table 2:** Demographic and disease-specific findings for entire cohort and individual study groups

Variable	Entire cohort (*n* = 71)	Normal-Mild (*n* = 58)	Moderate-Severe (*n* = 13)	P-value
Average age	59.7	60.4	59.7	0.79
Gender				
Males	50 (70.4 %)	46 (79.3 %)	8 (61.5 %)	0.65
Females	21 (29.6 %)	12 (20.7 %)	5 (38.5 %)	0.51
TNM staging				
Early	7 (9.9 %)	12 (20.7 %)	3 (23.1 %)	0.35
Advanced	64 (90.1 %)	46 (79.3 %)	10 (76.9 %)	0.32
Site				
Oral Cavity/Oropharynx	39 (54.9 %)	29 (50.0 %)	10 (76.9 %)	0.07
Larynx	9 (12.7 %)	9 (15.5 %)	0 (0.0 %)	0.11
Other	23 (32.4 %)	20 (34.5 %)	3 (23.1 %)	0.32
Reconstruction				
Osseocutaneous FF	11 (15.5 %)	9 (15.5 %)	2 (15.4 %)	1.00
Fasciocutaneous FF	58 (81.7 %)	48 (82.8 %)	10 (76.9 %)	0.24
Pedicled/Rotational	2 (2.8 %)	1 (1.7 %)	1 (7.7 %)	0.35
Adjuvant therapy				
Radiation therapy (RT)	57 (80.3 %)	46 (79.3 %)	11 (84.6 %)	0.56
Chemotherapy (C)	32 (45.1 %)	26 (44.8 %)	6 (46.2 %)	0.22
Chemoradiation Therapy (CRT)	32 (45.1 %)	26 (44.8 %)	6 (46.2 %)	0.22
Pre-treatment substance abuse	16 (22.5 %)	15 (25.9 %)	1 (7.7 %)	0.82
Supports/Caregivers available	61 (85.9 %)	48 (82.8 %)	11 (84.6 %)	0.81
Charlson comorbidity index	Mean 70.2 %, Median 77 %	Mean 70.2 %, Median 77 %	Mean 70.2 %, Median 90 %	0.65

*FF* free flap, *RT* radiation therapy, *CRT* chemoradiation therapy, *C* chemotherapy

At baseline 35.2 % of patients displayed Mild-only depressive symptoms and 18.3 % of patients displayed Moderate-Severe depressive symptoms. None of the patients expressed or recorded thoughts of suicidal ideation. More extensive evaluation of the domains of the QIDS questionnaire revealed significantly higher scores in the sleep, mood, appetite, concentration, energy level, and psychomotor domains for the Moderate-Severe group, indicating dysfunction in these specific areas (Table 3).

**Table 3:** Mean QIDS-SR scores at baseline and 12 months postoperatively for Normal-Mild and Moderate-Severe groups

	Mean baseline QIDS-SR	Mean 12-Month QIDS-SR	P-value
Normal-Mild	5.35	8.13	0.67
Moderate-Severe	14.8	17.1	0.58

*QIDS-SR* Quick Inventory of Depressive Symptomatology-Self Report

There were no statistically significant differences between baseline and 12-month QIDS scores in the Normal-Mild (*p* = 0.67), or Moderate-Severe groups (*p* = 0.58) (Table 4). FACT-HN scores at the 12th post-operative month demonstrated a statistically significant difference between subjects in the Normal-Mild and Moderate-Severe groups (*p* = 0.03). A multiple regression analysis was performed including other known and collected predictors of PFPS, which demonstrated Moderate-Severe PDS as a statistically significant predictor of postoperative FACT-HN scores (Table 5). The risk ratio for FACT-HN score less than 50 % with Moderate-Severe PDS was calculated to be 5.66.

**Table 4:** Baseline preoperative depressive symptoms (PDS) demonstrating significant differences between Normal-Mild and Moderate-Severe groups in 6 of 9 domains on the QIDS-SR

Preoperative Depressive Symptom (PDS)	P-value
Sleep	0.03*
Mood	0.01*
Appetite	0.01*
Concentration	0.01*
Self-Worth	0.61
Death-Suicide	0.61
Interest	0.10
Energy level	<0.01*
Psychomotor	<0.01*

*denotes statistical significance

**Table 5:** Multiple regression analysis including other known predictors of PFPS as measured on the FACT-HN, demonstrating severity of PDS as a predictor of PFPS

Variable	P-value
Age	0.37
Gender	0.38
Social Supports	0.77
Advanced Stage	0.76
Charlson Comorbidity Index	0.73
Moderate-Severe Preoperative Depressive Symptoms (PDS)	0.03*

*denotes statistical significance

Analysis of the secondary outcomes revealed a significantly lower rate of completion of adjuvant therapy in the Moderate-Severe group when compared to the Normal-Mild group (*χ*2 = 6.1, *p* = 0.03). A statistically higher rate of narcotic dependence was found in the Moderate-Severe group (*χ*2 = 8.8, *p* < 0.01). Higher rates of return-to-habit status were identified in the Moderate-Severe group (50 %) as compared to the Normal-Mild group (10.7 %). These results were not significant using a Fisher Exact analysis, though a trend was evident (*χ*2 = 3.7, *p* = 0.05). No statistically significant difference was noted between groups regarding loss of follow-up (*χ*2 = 0.67, *p* = 0.64) (Table 6). The mean LOHS for patients in the Moderate-Severe group was significantly longer than the Normal-Mild group Normal-Mild group (18 vs 24 days, *p* = 0.02).

**Table 6:** Relationship between PDS and secondary outcomes using Fisher Exact analysis

Variable	P-value
Completion of adjuvant therapy	0.01*
Narcotic dependence	<0.01*
Return-to-habit	0.05
Loss of Follow-up	0.64

*denotes statistical significance

**Table 7:** Outcomes and interventions of Moderate-Severe HNC group

Patient	Intervention	Status
1	Antidepressant	Deceased
2	Antidepressant	Living
3	Antidepressant	Deceased
4	Refused antidepressant; anxiolytic	Deceased
5	Antidepressant + anxiolytic	Deceased
6	Antidepressant + anxiolytic	Living; recurrence
7	Antidepressant + anxiolytic	Living
8	Refused antidepressant; anxiolytic	Living; recurrence
9	Antidepressant	Living
10	Antidepressant	Living
11	Refused antidepressant; anxiolytic	Deceased
12	Antidepressant + anxiolytic	Living; recurrence
13	Antidepressant + anxiolytic	Living; recurrence

**Fig. 1:**
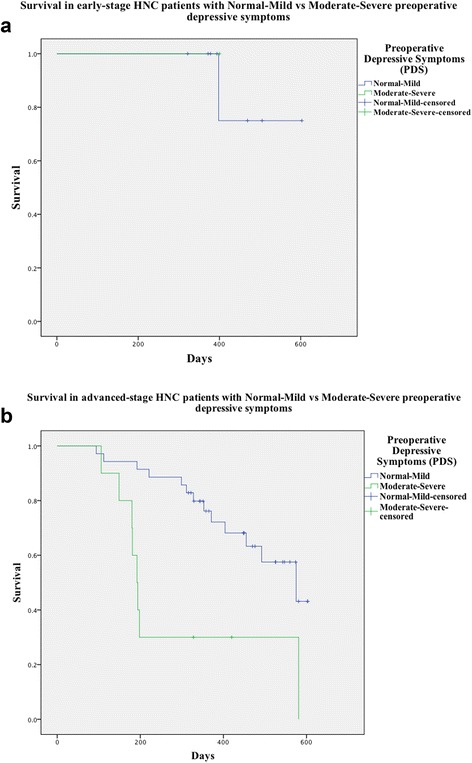
Kaplan-Meier curves illustrating disease-specific and overall survival (identical) for the Normal-Mild and Moderate-Severe groups after 12 months for: **a**) early-stage (Stage 1 and 2) and, **b**) advanced-stage (Stage 3 or 4) HNCs

Interventions for patients with Moderate-Severe symptoms are summated in Table 7. Of the patients in the Moderate-Severe group, 5 (38.5 %) were deceased at 12 months, and 4 (30.8 %) were living with recurrence. In the Normal-Mild group, 21 patients (30.0 %) were living with recurrence at 12 months, thus no difference in recurrence was detected between groups at 12 months (*χ*2 = 2.1, *p* = 0.22). However, both disease-specific (DSS) and overall survival (OS) were statistically significantly worse in the Moderate-Severe group at 12 months (*p* = 0.00, *p* = 0.00) (Table 8, Fig. 1). No non-cancer-related deaths occurred within 12 months of follow-up.

**Table 8:** Locoregional recurrence, disease-specific survival, and overall survival in Normal-Mild and Moderate-Severe groups 12 months post-treatment

Variable	Entire cohort (*n* = 71)	Normal-Mild (*n* = 58)	Moderate-Severe (*n* = 13)	p-value
Recurrence	25 (35.2 %)	21 (36.2 %)	4 (30.8 %)	0.22
Disease-Specific Survival (DSS)	59 (83.1 %)	51 (87.9 %)	8 (61.5 %)	<0.01
Overall Survival (OSS)	51 (87.9 %)	51 (87.9 %)	8 (61.5 %)	<0.01

*DSS* disease-specific survival, *OS* overall survival

## Discussion

This study demonstrates that the baseline prevalence and severity of preoperative depressive symptoms is high (53.5 %) in HNC patients, and that moderate or severe preoperative depressive symptoms is associated with lower overall postoperative functional performance status, higher rates of narcotic dependence, decreased treatment adherence, and a longer length of hospital stay. This relationship is independent of demographic factors, tumor site, TNM staging, surgical reconstruction, type or presence of adjuvant treatment, and medical comorbidities. These preliminary findings suggest that depressive symptoms in HNC patients impart significant effects on post-treatment rehabilitation and potentially overall survival.

Postoperative functional performance status (PFPS) has broad implications for postoperative course in HNC patients, given the extensive rehabilitation required for swallowing, speech, wound/stoma maintenance, and upper extremity physiotherapy. Moreover, functional status can impact ability to physically attend adjuvant treatments, and this can be compounded by depressive symptoms. As such, PFPS and depressive symptoms alike should be examined as contributors to survival, given their association with low treatment adherence in other types of cancer [9, 10]. This study demonstrates a significantly lower post-treatment functional status in patients displaying Moderate-Severe PDS, with a RR of 5.66 in obtaining a score less than 50 % on the FACT-HN questionnaire 12 months postoperatively. A similar study by Lin et al. [11] examined the relationship between severe depressive symptoms and specific swallowing and speech outcomes using the MD Anderson Dysphagia Inventory (MDADI) and Beck Depression Inventory Fast Screen (BDI-FS), and found significantly lower MDADI scores for depressed patients 1 year post-treatment. This study also demonstrated a lower overall quality of life (QOL) in patients with lower BDI –FS scores at this time interval. Although it was not our express intention to examine QOL with increasing severity of PDS, previous studies have correlated decreasing FACT-HN scores with decreasing QOL, which in turn has been shown to be a significant prognostic factor in HNC survival [12].

Narcotic dependence was found to be significantly associated with PDS (*p* = 0.004). In the Moderate-Severe group, 4 of the 8 patients remaining alive had recurrence, which may have contributed to the increased incidence of narcotic dependence. However, there was no significant difference between the Normal-Mild and Moderate-Severe groups regarding recurrence at 12 months, thus this confounder should be eliminated. The relationship between narcotic dependence, pain, PDS, and PFPS is not clear. It is possible that patients with more severe PDS have chronic pain throughout the treatment process that contributes to a decreased PFPS. A previous study by Shuman et al. [13] showed that severe depressive symptoms were a significant predictor of pain in head and neck cancer patients 1-year post-treatment. Conversely, other authors [14] have demonstrated an inverse relationship wherein pain is a predictor of depression in post-treatment cancer survivors. Therefore, it can be inferred that these symptoms can occur in parallel, and should be monitored throughout diagnosis and treatment.

There was a near-significant relationship between PDS and a return to detrimental habits in our HNC population (*p* = 0.05). There is a well-established relationship between addictions and mental illness, and thus, this relationship, although marginal in this study, is not unexpected. No significant difference in pre-treatment substance abuse was detected between groups that could have been considered to contribute to QIDS scores or even treatment adherence. A recent study by Berg et al. [15] demonstrated that, among survivors of all smoking-related cancers, severe depressive symptoms were a significant risk factor for continued tobacco use, yet it is not known how much this relationship may contribute to survival and recurrence in this population of patients. A previous study by Jerjes et al. [16] demonstrated a significant reduction in mortality at 3 and 5 years with alcohol and tobacco cessation when compared to patients engaging in persistent use. Surveillance and treatment for severe depressive symptoms is potentially warranted, given the tendency of HNC patients for relapse into detrimental habits and the impact of this on survival.

Perhaps the most compelling finding in this study is a significant reduction in treatment adherence by patients in the Moderate-Severe study group. While it is known that substance abuse is common in the HNC population, and may therefore contribute to decreased treatment adherence, our results demonstrated that there was no difference in preoperative substance abuse between groups, yet treatment adherence was significantly worse in the Moderate-Severe group. A previous study by Lazure et al. [17] demonstrated that HNC patients with a diagnosis of major depressive disorder (MDD) have a 25 % greater mortality than non-depressed patients, independent of TNM staging. However, the cause for this significant reduction in survival was not clear. A multi-factorial explanation is probable, given the complexity of treatment modalities and postoperative rehabilitation in HNC. Failure of completion of adjuvant therapy is certain to contribute to this mortality rate, as it is known that timely completion of radiation therapy is an important predictor of successful disease control [18–20]. Our study demonstrated a significantly lower disease-specific (DSS) and overall survival (OS) in the Moderate-Severe group, however with only 12 months of follow-up. This suggests that any potential contribution to decreased survival rates imparted by depressive symptoms likely affects patients early, and potentially in the capacity of completion of adjuvant therapy. However, longer follow-up and further study is required to make this association.

The limitations of this study are its relatively small sample size and single-institution status. Future studies should aim to examine this condition in large cohorts in a multi-institutional manner. In addition, this was an exclusively surgical cohort of patients. We elected to include only surgical patients in our study due to the unique nature of their cosmetic concerns postoperatively and the potential differences in symptoms they may have compared to non-surgical patients. Future study should consider non-surgical patients to determine differences or similarities in optimal treatment regimens for both groups. As well, patients with “Mild” symptoms were included in analysis with “Normal” patients. This decision was made in psychiatric consult, as it was reasoned that the clinical manifestations of Moderate-Severe depression would be more likely to cause functional disability, and also be the cutoff for consideration of treatment. Lastly, this study involves relatively short follow-up of 12 months. This endpoint was chosen given the “acute” nature of the first 12 months after treatment, and the fact that often, 12 months postoperatively is often when patients consider a return to work. In addition, stated survival results are done so with cautionary connotation, as they apply in the context of 12-month follow-up. Continued study of the functional and survival status of the cohort is ongoing, and a screening and treatment algorithm has been integrated into the HNC clinical care pathway at the University of Alberta to ensure sustained progress.

## Conclusions

The prevalence of preoperative depressive symptoms is high in HNC patients. The effect of PDS on post-treatment functional status and rehabilitation, as well as treatment adherence, can act as significant contributing factors in postoperative course, and given these findings, early screening and intervention to avert the effects of moderate or severe depressive symptoms on postoperative rehabilitation should be considered.

## Abbreviations

HNC: Head and neck cancer
PFPS: Postoperative functional performance status
PDS: Preoperative depressive symptoms:
QIDS: Quick Inventory of Depressive Symptoms
FACT-HN: Functional Assessment of Cancer Therapy for Head & Neck Cancer patients
RTOG: Radiation Therapy Oncology Group
CCI: Charlson Comorbidity Index
MDADI: MD Anderson Dysphagia Index
BDI-FS: Beck Depression Inventory Fast Screen
DSS: Disease-specific survival
OS: Overall survival
